# *Shamba Maisha*: Pilot agricultural intervention for food security and HIV health outcomes in Kenya: design, methods, baseline results and process evaluation of a cluster-randomized controlled trial

**DOI:** 10.1186/s40064-015-0886-x

**Published:** 2015-03-12

**Authors:** Craig R Cohen, Rachel L Steinfeld, Elly Weke, Elizabeth A Bukusi, Abigail M Hatcher, Stephen Shiboski, Richard Rheingans, Kate M Scow, Lisa M Butler, Phelgona Otieno, Shari L Dworkin, Sheri D Weiser

**Affiliations:** Department of Obstetrics, Gynecology & Reproductive Sciences, University of California San Francisco, 550 16th Street, San Francisco, CA 94158 USA; Departments of Medicine, University of California San Francisco, San Francisco, CA USA; Departments of Epidemiology and Biostatistics, University of California San Francisco, San Francisco, CA USA; Departments of Social and Behavioral Sciences, University of California San Francisco, San Francisco, CA USA; Center of Expertise in Women’s Health & Empowerment, University of California Global Health Institute, San Francisco, CA USA; Centre for Microbiology Research, Kenya Medical Research Institute, Nairobi, Kenya; Centre for Clinical Research, Kenya Medical Research Institute, Nairobi, Kenya; Department of Environmental and Global Health, University of Florida, Gainesville, FL USA; Department of Soil Science and Soil Microbial Biology, University of California Davis, Davis, CA USA; Wits Reproductive Health and HIV Institute, University of the Witwatersrand, Johannesburg, South Africa; Boston Children’s Hospital and Harvard Medical School, Boston, MA USA

**Keywords:** HIV, Food insecurity, Microfinance, Agriculture, Livelihoods, Intervention

## Abstract

**Background:**

Despite advances in treatment of people living with HIV, morbidity and mortality remains unacceptably high in sub-Saharan Africa, largely due to parallel epidemics of poverty and food insecurity.

**Methods/Design:**

We conducted a pilot cluster randomized controlled trial (RCT) of a multisectoral agricultural and microfinance intervention (entitled *Shamba Maisha*) designed to improve food security, household wealth, HIV clinical outcomes and women’s empowerment. The intervention was carried out at two HIV clinics in Kenya, one randomized to the intervention arm and one to the control arm. HIV-infected patients >18 years, on antiretroviral therapy, with moderate/severe food insecurity and/or body mass index (BMI) <18.5, and access to land and surface water were eligible for enrollment. The intervention included: 1) a microfinance loan (~$150) to purchase the farming commodities, 2) a micro-irrigation pump, seeds, and fertilizer, and 3) trainings in sustainable agricultural practices and financial literacy. Enrollment of 140 participants took four months, and the screening-to-enrollment ratio was similar between arms. We followed participants for 12 months and conducted structured questionnaires. We also conducted a process evaluation with participants and stakeholders 3–5 months after study start and at study end.

**Discussion:**

Baseline results revealed that participants at the two sites were similar in age, gender and marital status. A greater proportion of participants at the intervention site had a low BMI in comparison to participants at the control site (18% vs. 7%, p = 0.054). While median CD4 count was similar between arms, a greater proportion of participants enrolled at the intervention arm had a detectable HIV viral load compared with control participants (49% vs. 28%, respectively, p < 0.010). Process evaluation findings suggested that *Shamba Maisha* had high acceptability in recruitment, delivered strong agricultural and financial training, and led to labor saving due to use of the water pump. Implementation challenges included participant concerns about repaying loans, agricultural challenges due to weather patterns, and a challenging partnership with the microfinance institution. We expect the results from this pilot study to provide useful data on the impacts of livelihood interventions and will help in the design of a definitive cluster RCT.

**Trial registration:**

This trial is registered at ClinicalTrials.gov, NCT01548599.

**Electronic supplementary material:**

The online version of this article (doi:10.1186/s40064-015-0886-x) contains supplementary material, which is available to authorized users.

## Background

Despite major advances in care and treatment of those living with HIV, morbidity and mortality among people living with HIV/AIDS (PLHIV) remains unacceptably high in sub-Saharan Africa, largely due to parallel epidemics of poverty and food insecurity (Weiser et al. 2011). There are an estimated 35.3 million PLHIV worldwide, 70.8% of whom live in sub-Saharan Africa (Global report 2013). Food insecurity, defined as “the limited or uncertain availability of nutritionally adequate, safe foods or the inability to acquire personally acceptable foods in socially acceptable ways,” (Normen et al. 2005) is also highly prevalent in sub-Saharan Africa, where 240 million persons, or one in every four people, are estimated to be food insecure (FAO 2010). The prevalence of food insecurity is even higher among PLHIV in sub-Saharan Africa. Studies from Kenya and Uganda have shown that over 50% of PLHIV are moderately or severely food insecure (Mbugua et al. 2008; Weiser et al. 2010a).

Food insecurity and HIV/AIDS are leading causes of morbidity and mortality in sub-Saharan Africa and are inextricably linked, with each condition heightening vulnerability to, and worsening the severity of the other condition (Weiser et al. 2011). Food insecurity enhances HIV acquisition risk through increased risky sex and also increases susceptibility to HIV among those who are exposed (Weiser et al. 2007; Campbell et al. 2009; Webb-Girard et al. 2012; Weiser et al. 2011). Among PLHIV, food insecurity inhibits antiretroviral therapy (ART) initiation, retention in care, and ART adherence (Weiser et al. 2010b; Goudge & Ngoma 2011; Nagata et al. 2012; Weiser et al. 2009a; Wang et al. 2011; McMahon et al. 2011; Weiser et al. 2009b; Weiser et al. 2012). Food insecurity has been associated with a range of adverse clinical effects among PLHIV, including declines in physical health status (Weiser et al. 2009c; Weiser et al. 2012), decreased viral suppression (Weiser et al. 2009a; Kalichman et al. 2010a), worse immunologic status (Weiser et al. 2009c; Kalichman et al. 2010b), increased incidence of serious illness (Tsai et al. 2011), and increased mortality (Weiser et al. 2009b). In turn, HIV/AIDS worsens food insecurity by eroding economic productivity (Larson et al. 2008; McIntyre et al. 2006; Russell 2004), reducing social support due to HIV stigma (Tsai et al. 2011), and increasing medical expenses (McIntyre et al. 2006).

Although there has been a substantial increase in the allocation of international resources towards HIV care and treatment programs in Africa, food insecurity can significantly compromise the effectiveness of these programs due to its effects on morbidity and mortality as described above (Mamlin et al. 2009). As a result, the World Health Organization, UNAIDS and the World Food Programme have recommended integrating sustainable food production strategies into HIV/AIDS programming (Food and Nutrition Technical Assistance. HIV/AIDS: A Guide For Nutritional Care and Support; Nutrition and HIV/AIDS 2001; Blumberg & Dickey 2003; World Food Program 2003). Specifically, UNAIDS calls for international partners to “fund multisectoral HIV programming that incorporates effective food and nutrition interventions, in line with scale-up towards universal access to prevention, treatment, care and support” (UNAIDS Policy Brief 2008). Yet, little research exists to document the beneficial effect of food security or sustainable agricultural interventions on antiretroviral (ARV) adherence, HIV clinical outcomes, women’s empowerment and HIV transmission risk behaviors among PLHIV in Africa or elsewhere.

To date, no randomized controlled trials have been conducted in resource-limited settings to examine the impacts of either food supplementation or sustainable food production strategies on HIV morbidity and mortality (Mahlungulu et al. 2007). Several small studies in developing countries have demonstrated the potential for programs that address food security to affect health outcomes among PLHIV (Mamlin et al. 2009; Cantrell et al. 2008; Agricultural initiatives for health in Haiti; Ochai 2008; Njenga et al. 2009; Byron et al. 2008). Yet, existing intervention approaches to impacting food security have focused primarily on direct macronutrient supplementation, which may be somewhat limited in its scalability and sustainability (Sztam et al. 2010). Livelihood interventions, which address upstream causes of food insecurity, may have a better chance of improving health outcomes, and may be more sustainable. Similarly, while microcredit programs can improve health and prevent disease acquisition by targeting poverty and gender inequality (Ashburn et al. 2008; Kim et al. 2008; Schuler & Hashemi 1994), they have been criticized in terms of their effectiveness as a stand-alone strategy. As a result, experts have recommended integrating microfinance and other livelihood approaches to maximize HIV prevention and treatment efforts and reduce poverty (Dworkin & Blankenship 2009; Weinhardt et al. 2009). Income generating activities are well suited to improving food security (Diagne 1998; Doocy et al. 2005), and to retaining patients in HIV care (Mamlin et al. 2009; Gomez et al. 2004).

Following our previously published theoretical model (Weiser et al. 2011), we set out to test the impact of a multisectoral agricultural intervention on HIV health outcomes and transmission risk behaviors in rural Kenya. In a previous small feasibility study conducted by our group, we showed that using a human powered irrigation hip pump combined with a microfinance loan led to increases in crop yields, household income, CD4 counts and BMI (Pandit et al. 2010). In the current study, we developed and tested a modified version of this combination agricultural and microfinance intervention called *Shamba Maisha,* Kiswhahili for “farm life,” in a small community randomized control trial. In this study, we aimed to explore the acceptability and feasibility of the intervention and control conditions that will be used in a subsequent, larger cluster randomized controlled trial, and examined preliminary impacts on outcomes of interest. Two similar district hospitals in southern Nyanza Province were randomized: one to the intervention and the other to the control group. We hypothesized that this multisectoral intervention will improve food insecurity, household wealth and HIV health outcomes. We developed and tested a theoretical framework for the pathways through which this multisectoral agricultural intervention may improve health. In this paper, we described our conceptual framework, study methods, baseline findings, as well as process evaluation findings of successes and challenges with implementation.

## Methods

### Setting

The study took place in Rongo and Migori districts in Nyanza Province, Kenya. As of 2008, the HIV prevalence in Nyanza Province, Kenya, was estimated to be 15.3%, more than twice the national average (Kenya National Bureau of Statistics and ICF Macro 2010). Nearly all HIV-affected households in a recent Kenyan survey were considered to be moderately or severely food insecure (Mbugua et al. 2008). The province also has a significant shortage of accessible water making communities vulnerable to the impacts of drought, and a heavy dependence on an unstable agricultural sector. Farming and fishing are the primary means of income generation in Nyanza Province. Lack of irrigation and unpredictable rainfall leading to an inconsistent water supply remains a central barrier to successful farming for many in the region (Government of Kenya 2008).

### Description of intervention

The *Shamba Maisha* intervention consisted of three components: 1) a microfinance loan (~$150) to purchase the farming commodities, 2) a micro-irrigation pump, seeds, fertilizer and pesticides, and 3) trainings in sustainable agricultural practices and financial literacy. KickStart, an international non-governmental organization (NGO), along with technical support of agricultural experts from the University of California Davis, led the agricultural component of the intervention. *Adok Timo*, a microfinance institution in Nyanza Province, implemented the economic aspects of the intervention with support from the UCSF and KEMRI research team. The intervention components are described below.

#### Loan program

The microfinance loans were managed by *Adok Timo,* which has branches in Nyanza Province. The intervention group received training on financial management and marketing skills prior to receiving the loan. All trainings took place on participant’s farms or a nearby location. Participants were required to save 500 Kenyan shillings (~$6.00 USD) prior to receiving the loan. Each participant received vouchers to purchase the following items: the Hip Pump, 50 feet of hosing, fertilizer, and government certified seeds. These materials were purchased at a local farm store (“agrovet”). Loan repayment could begin at any time, but farmers were expected to make a minimum payment after the first harvest, usually 4–6 months after planting. Farmers were expected to repay in full by the end of two harvest seasons (approximately one year); however, if regular payments have been made, this deadline was extended based on the guidelines set out by the microfinance institution. Participants were not asked to forfeit personal belongings to cover loan payments. Control participants were eligible for the microcredit loan and the *Kickstart* Hip Pump at the end of the 1-year follow-up period.

KickStart water pump and agricultural training: Irrigation technologies have been shown to improve agricultural output. (About KickStart) Recognizing the need for improved agricultural tools for poor farmers in Kenya, Kickstart developed a low-cost irrigation pump. These pumps enable farmers to irrigate their crops year-round avoiding dependence on seasonal rainfall thus capitalizing on higher crop prices in the marketplace. In prior evaluations in Kisumu Kenya, farmers using this hip pump have been able to enjoy up to a ten-fold increase in income (Brandsma 2003; Kihia & Kamau 1999; Stevens 2002; World Bank 2010).

Prior to receiving the microfinance loan, participants in the intervention group received eight training modules. The training modules were delivered to groups of farmers on participant’s farms or at a nearby location by agricultural trainers from Kickstart, the loan officer from *Adok Timo*, and the study coordinator. The agricultural portion of the training included a didactic session and practical demonstrations on sustainable farming techniques, seed selection, soil and water conservation, fertilization and crop rotation, integrated pest and disease management (IPM), pre & post-harvest handling and marketing, the use of the MoneyMaker Hip Pump, and identifying improved market access for selling horticultural products. The field-based practical trainings were based on a training needs assessment and the crops selected by the participants. These trainings focused on plant spacing, seed selection, seed rate, crop combination, and a cost-benefit analysis for each crop selected and IPM. Participants were particularly interested in locally available materials for IPM like wood ash, Mexican marigold and livestock urine in controlling pests on crops to enable them to reduce cost and maximize profits. Participants also conducted a market survey as part of the pre & post-harvest handling and marketing which revealed a large demand in the local markets for green vegetables and watermelon. The intervention participants also had an in-depth training on financial record keeping, the importance on savings and loans, and the basics of group dynamics.

### Intervention model

The primary aim of the pilot study was to test if a multisectoral agricultural intervention improves food security and HIV health outcomes. We hypothesized that the proposed multisectoral agricultural intervention will improve food security, prevent treatment failure, reduce co-morbidities, and decrease secondary HIV transmission risk among PLHIV.

**Figure 1:**
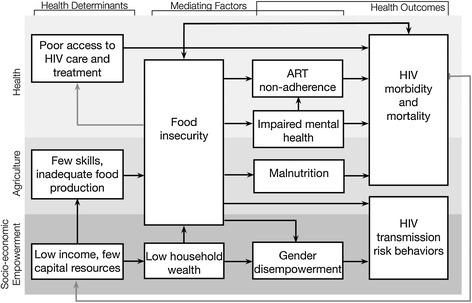
***Shamba Maisha*** **causal framework**

We developed an evidenced-based causal framework (Figure 1) to understand the pathways by which food insecurity negatively impacts health outcomes. Food insecurity negatively impacts health outcomes through nutritional, behavioral, and mental health pathways, which emerge directly from the definition of food insecurity (Weiser et al. 2011). In terms of nutritional pathways, food insecurity has been associated with macronutrient and micronutrient malnutrition (Rose & Oliveira 1997; Lee & Frongillo 2001), and weight loss, low body mass index (BMI), and low albumin have been shown to hasten progression to AIDS and death (Stringer et al. 2006; Zachariah et al. 2006; Johannessen et al. 2008). HIV also increases metabolic requirements (Babameto & Kotler 1997; Macallan et al. 1995) and is associated with diarrhea and malabsorption of fat and carbohydrates (Babameto & Kotler 1997; Kotler et al. 1990; Stack et al. 1996; Fields-Gardner & Fergusson 2004). Lack of food may also impede optimal absorption of certain ARVs (Gustavson et al. 2000; Bardsley-Elliot & Plosker 2000; Food and Agriculture Organization 2010), which may contribute to treatment failure. In terms of behavioral pathways: Food insecurity is an important cause of ART non-adherence and treatment interruptions, (Weiser et al. 2010b; Weiser et al. 2009a; Kalichman et al. 2010a; Weiser et al. 2009d); Tuller et al. 2010) which are both well-known determinants of HIV treatment outcomes (Parienti et al. 2004; Parienti et al. 2008; Oyugi et al. 2007). In addition to ART non-adherence and treatment interruptions, as a result of competing demands between food and other resources, food insecure individuals often miss scheduled clinic visits, and may be less likely to initiate ART (Weiser et al. 2010b; Tuller et al. 2010; Weiser et al. 2012). In terms of mental health pathways, food insecurity has been associated with depression and poor mental health status in sub-Saharan Africa and elsewhere, (Weiser et al. 2009c; Tsai et al. 2011; Anema et al. 2011) which in turn have been shown to independently contribute to lower ART adherence and worse HIV clinical outcomes (Ickovics et al. 2001; Tucker et al. 2003; Evans et al. 2002; Weiser et al. 2004). Food insecurity and poverty also contribute to lower levels of empowerment, including women’s empowerment, which can negatively impact health outcomes for HIV. For example, in Uganda, among HIV-infected women low sexual relationship power is associated with malnutrition (Siedner et al. 2012), depression (Hatcher et al. 2012), and worse virologic outcomes (Weiser et al. 2010a, b).

**Figure 2:**
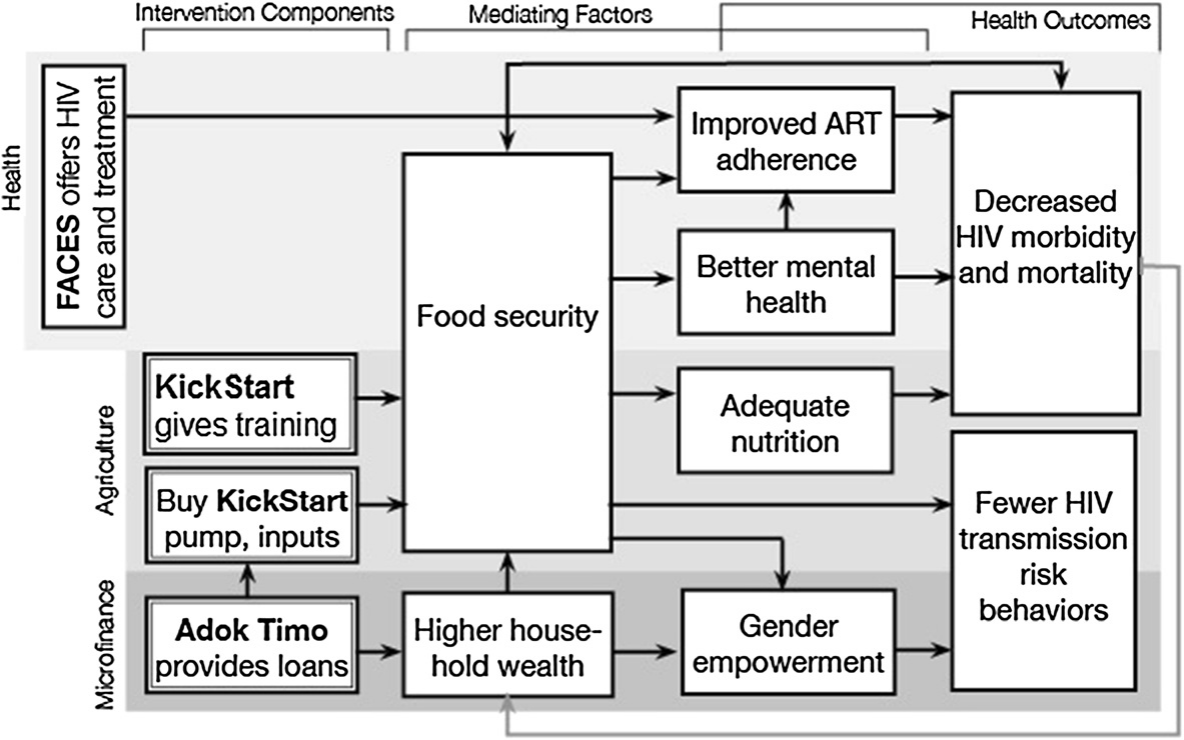
***Shamba Maisha*** **intervention framework**

Drawing upon this framework, we developed the intervention framework to describe the pathway by which we believe the multisectoral agrigultural intervention impacts on mediating factors and eventually improves health outcomes among HIV-infected adults (Figure 2). Specifically, we hypothesized that: 1) The three intervention components together would directly lead to improvements in food security and household wealth (most proximal mediators); 2) Changes in food security and household wealth would, in turn, contribute to less macronutrient and micronutrient malnutrition (nutritional pathway), less anxiety, stress and depression (mental health pathway), improved ART adherence and retention in care (behavioral pathway) and improved gender empowerment; and 3) Through these pathways, the intervention would ultimately contribute to decreased HIV morbidity and mortality and fewer HIV transmission risk behaviors.

### Randomization

We used a two-step process to select two sites from among 68 government health facilities in Rongo, Migori and Nyatike districts supported by Family AIDS Care & Education Services (FACES), a collaboration between the University of California, San Francisco (UCSF), and the Kenyan Medical Research Institute (KEMRI) (Lewis Kulzer et al. 2012). First, we limited to 20 sites where there were an adequate number of PLHIV on ART at the time of the study, and where farming was a primary means of livelihood in the communities. Next, we selected two government health facilities for the pilot randomized controlled trial (RCT) according to the following criteria: a) having adequate numbers of PLHIV on ART with non-overlapping catchment areas; b) having catchment areas that were relatively similar to one another according to rainfall patterns, health, topography and socioeconomic status; and c) meeting practical requirements for the intervention, including acceptable characteristics in topography to enable use of the water pump, having water access year round, and having favorable soil composition to potentiate the intervention. One site was randomly selected as the intervention site and the other the control site by the study’s biostatistician who used a computer random number generator and was not involved in fieldwork. Clinics, healthcare providers, patients, and researchers involved in implementing the study were not blinded to the allocation.

### Ethics statement

The study was approved by the Committee on Human Research at UCSF and the Ethical Review Committee at KEMRI. All participants in the study gave written informed consent prior to enrollment in the study, and had the cost of their transportation reimbursed up to 800 Ksh per clinic-based interviews (~$9.4 USD) and 400 Ksh for home-based interviews (~$4.70). This clinical trial was registered at ClinicalTrials.gov (NCT01548599).

### Participants and recruitment

**Figure 3:**
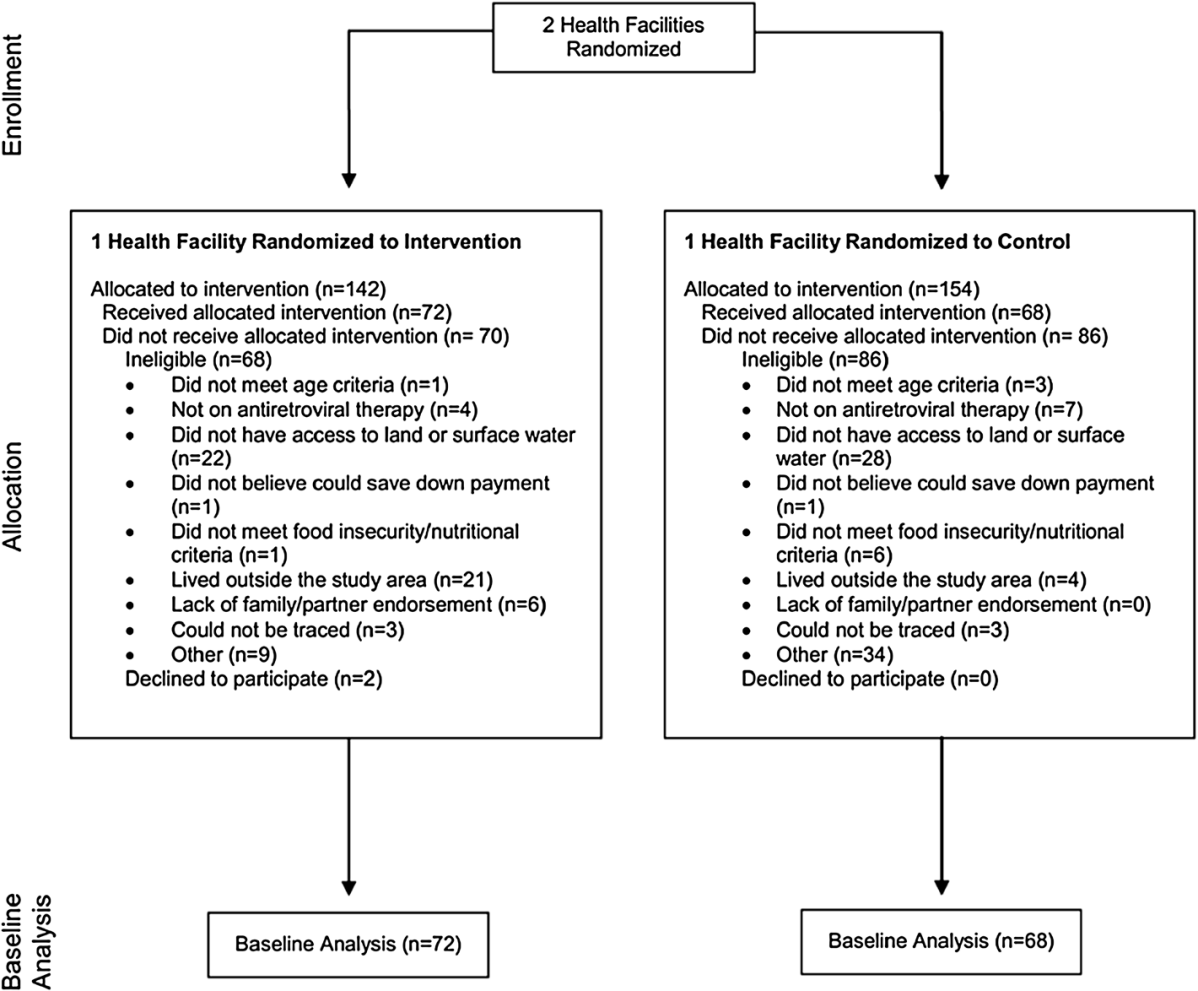
***Shamba Maisha*** **consort diagram**

The study population includes HIV-infected patients between the ages of 18–49 years receiving ART, have access to farm land and surface water, and have demonstrated evidence of moderate to severe food insecurity or malnutrition during the year preceding the study. Participants were recruited from Rongo (intervention site) and Migori (control site) District Hospitals in Nyanza Province beginning in April 2012. Research assistants introduced the study to patients waiting to be seen at the HIV clinic at the two health facilities. Individuals who expressed interest were consented and screened for eligibility. Among interested and potentially eligible individuals, home visits were conducted to verify that the participant had access to farming land and surface water. In addition to the eligibility criteria above, the study also aimed to ensure that at least 40% of participants at each site were of each gender. Individuals who met eligibility criteria were enrolled in the study after providing written informed consent. At intervention sites, participants were enrolled in a savings program in anticipation of receiving the asset loan. Control participants also agreed to save the down payment (~$6) required for the loan by the study end. A total of 140 HIV-positive individuals were enrolled in the study during the period of April – July 2011 (See Figure 3); an additional two (1.4%) eligible screened participants declined to participate in the study.

### Data collection

Participants were followed quarterly for structured interviews and ART adherence assessments by unannounced pill counts at their home. Anthropometry (i.e., mid upper arm circumference measurements [MUAC] and BMI measurements) and phlebotomy for viral load and CD4 determinations were conducted every six months. Viral load testing was performed on venous blood at the Centers for Disease Control-Kenya laboratory on the COBAS TaqMan HIV viral load platform (Roche Molecular Diagnostics, Pleasanton, CA) with a lower limit of detection of <20 copies/mL. Absolute CD4 count testing was performed on whole blood using the BD FACSCount (BD Bioscience, San Jose, CA). In addition, clinical data were abstracted from participant’s medical records.

Structured interviews for clinical and sensitive behavioral data were administered at baseline, 6 months and 12 months at the clinic and included the following data (see Table 1): health care access and competing demands, ARV history and adherence, physical and mental health, social support, women’s empowerment, stigma, HIV disclosure, alcohol use, and sexual behavior. Additionally, quarterly home visits were conducted to collect unannounced pill counts for ART adherence, food frequency, food security, income, housing, assets and wealth, and specific data related to agricultural output, labor, harvesting, marketing, and irrigation.

**Table 1:** **Measurements**

**Path**	**Measurement**	**Definition**	**Frequency**
**Nutritional Pathway**	Nutritional status	Nutritional status was assessed through body mass index (BMI) and mid-upper arm circumference (MUAC), commonly used to assess nutritional status (Physical Status 1995; Collins et al. 2000). The BMI reflects protein and fat reserves (James et al. 1988) and was assessed using an established grading system (Ferro-Luzzi et al. 1992). For MUAC, we used WHO sex-specific cut-offs of 22.0 cm for females and 23.0 cm for males with chronic energy deficiency (Doocy et al. 2005).	Semi-annually
	Food frequency	Food frequency, the number of different foods or food groups and the frequency consumed over a given reference period (Hoddinott & Yohannes 2002), as adapted from the World Food Programme Food Consumption Score was collected.	Quarterly
**Behavioral Pathway**	Pill count ART adherence	Participants received an unannounced visit to inventory medications and count pills (Bangsberg et al. 2001a; Bangsberg et al. 2001b; Bangsberg et al. 2000), a technique closely correlated with electronically monitored adherence, HIV viral load (Bangsberg et al. 2000) and progression to AIDS (Bangsberg et al. 2001c). The count of existing pills was reconciled with the participant’s pharmacy refill history to determine the percentage of pills not yet consumed.	Quarterly
	Competing demands	Questions were modified from Gelberg and Anderson’s Behavioral Model for Vulnerable Populations (Gelberg et al. 2000; Gelberg et al. 1997) to assess how often lack of food interferes with ability to procure drugs or visit the clinic.	Semi-annually
	Healthcare access	Utilization of health care services including hospitalizations and clinic visits over the preceding 6 months were collected.	Semi-annually
**Mental Health Pathway**	Mental health & depression	Mental health status was measured using the Medical Outcomes Study HIV Health Survey (MOS-HIV), a tool for assessing health-related quality of life (Wu et al. 1991) that has been validated in resource-limited settings (Chatterton et al. 1999; Mast et al. 2004). Depression was screened using the Hopkins Symptom Check-list for depression, a 15-item scale (Derogatis et al. 1974) which has been validated in sub-Saharan Africa (Bolton et al. 2004).	Semi-annually
	HIV-related stigma	We used the Internalized AIDS-Related Stigma Scale (Kalichman et al. 2009).	Semi-annually
	Disclosure of HIV status	We asked about disclosure of HIV status to partners, family members, friends, colleagues, and public. These questions were adapted from our previous studies in Uganda, Botswana and Swaziland (Weiser et al. 2007; Wolfe et al. 2008; Tsai et al. 2013).	Semi-annually
	Alcohol use	To measure alcohol use, we adapted the Alcohol Use Disorders Identification Test (AUDIT-C) indicators. The AUDIT-C is a 3-item alcohol screen that can help identify persons who are hazardous drinkers or have active alcohol use disorders.	Semi-annually
**Empowerment**	Gender empowerment	Empowerment indicators were adapted from a large cluster-randomized trial of an intervention including: greater challenges to established gender roles, communication with relationship partner about sexual matters in the prior 3 months, measures of financial decision-making, measures of attitudes towards gender roles and gender-based violence, and experience of controlling behavior by relationship partner in prior 3 months (Pronyk et al. 2006). In addition, we used the Sexual Relationship Power Scale (SRPS) (Pulerwitz et al. 2000), which conceptualizes sexual relationship power as a multi-dimensional construct consisting of relationship control and decision making dominance. The SRPS has been used successfully in observational research conducted in South Africa (Dunkle et al. 2004; Jewkes et al. 2010) and Uganda.(Weiser et al. 2010a, b) We also collected data on sexual victimization and perpetration in the prior 3 months.	Semi-annually
**Proximal Mediators**	Food insecurity	The Household Food Insecurity Access Scale (HFIAS) has been validated in eight countries (Coates et al. 2006a; Swindale & Bilinsky 2006; Frongillo & Nanama 2006; Coates et al. 2006b) and used successfully by our team in rural Uganda. (Weiser et al. 2010a; Tsai et al. 2011; Weiser et al. 2012; Tsai et al. 2012; Weiser et al. 2010a, b; Miller et al. 2011; Tsai et al. 2011)	Semi-annually
	Agricultural measures	Agricultural measures were adapted from outcome evaluations developed by Kickstart in Kenya and supplemented by outcome indicators found in an earlier pilot study and a rural assessment. These measures were designed to evaluate uptake and adoption, and to measure changes in agricultural practices including crop diversity and agricultural practices and production. In addition, we evaluated the effectiveness of the training, and specific topics within, so as to refine the training for the subsequent larger cluster-randomized trial.	Quarterly
	Household economic indicators	A modification of the World Bank Living Standards Measurement Study (LSMS) questionnaire (Grosh & Glewwe 1998) was used to measure: a) expenditures (food, health, education and productive investments); b) consumption (food and non-food); c) income (from agriculture and all sources); and d) inter-household commodity and cash transfers.	Quarterly
**Behavioral Outcome**	Risky sexual behaviors	The primary transmission risk outcome was unprotected sex. Other outcomes included: number of non-spousal/non-cohabiting sexual partners, sex-exchange (exchanging sex for money, food, or other resources) Dupas & Robinson 2010); Robinson & Yeh 2011).	Semi-annually
**Health Outcomes**	HIV-related mortality	Burial permits and information from family members were used to determine cause of death.	As needed
	Viral load suppression	Viral load testing was performed on venous blood on the COBAS TaqMan HIV viral load platform (Roche Molecular Diagnostics, Pleasanton, CA) with a lower limit of detection of <20 copies/mL.	Semi-annually
	CD4 Count	We abstracted data for CD4 counts from participant’s medical records. Absolute CD4 count testing was performed on whole blood using the BD FACSCount (BD Bioscience, San Jose, CA).	Semi-annually
	HIV morbidity	HIV morbidity was measured through key outcomes from the medical record. We abstracted data every 3 months for ART treatment interruptions and episodes of opportunistic infections. We also gathered self-report data on opportunistic infections and symptoms during structured interviews.	Quarterly/Semi-annually
	Physical health	Health status was measured using the MOS-HIV, a tool for assessing health-related quality of life (Wu et al. 1991) that has been validated in resource-limited settings (Chatterton et al. 1999; Mast et al. 2004).	Semi-annually
**Covariates**	Demographics	Age, religion, education, marital/partnership status, number of children and household census.	Baseline
	Social support	To measure social support, we adapted the Functional Social Support Scale (Antelman et al. 2001), a modified version of the Duke University-University of North Carolina Functional Support Questionnaire (Broadhead et al. 1988) consisting of questions related to perceived emotional and instrumental support. Higher scores reflect higher levels of social support.	Semi-annually
	ART history and Self-reported ART adherence	Detailed ART history, ART self-reported adherence and barriers to ART adherence were collected. For self-report adherence, we used the visual analog scale (Oyugi et al. 2004) and the three day recall.	Semi-annually

### Data collection methods

Structured interviews administered at the health facility and at participants’ farms or homes were conducted by trained research assistants who used a handheld computer tablet for data entry (Morotola™ Xoom Android Tablets operating Open Data Kit (ODK) Collect (Hiarlaithe et al. 2014). Research assistants conducted structured interviews in the local language (Dholuo) or English and completed paper-based data collection forms for the majority of baseline data collection (Table 1). Forms were later entered into the tablet using ODK Collect. Research assistants entered subsequent data directly into the tablet. Data for anthropometry (MUAC and BMI), and viral load results were entered into ODK Collect forms (Table 1). Medical history and CD4 determinations were abstracted from patient records and entered into the tablet (Table 1).

### Monitoring

The study team conducted internal monitoring such as form completion and quality control of data entry monthly. Procedures to promote data quality including range and logical checks were built into the data entry program, and we ran a series of additional error checks on the databases following data entry. In addition, we used a rainfall gauge at the two study sites to record total precipitation by month.

### Process evaluation methods

In order to understand the implementation successes and challenges, we conducted a detailed process evaluation alongside the *Shamba Maisha* trial. We interviewed 40 intervention participants and 20 key informants at two time points (3–5 months after study start and study end) to understand successes and challenges with implementation (for a total of n = 120 interviews). Interviews were conducted in English, Dhuluo or Kiswahili, and lasted between 45 minutes and 2 hours. We used a semi-structured interview guides to probe for feedback on recruitment and retention as well the key intervention components (the microcredit loan, the agricultural training, and the use of the hip pump), and elicit suggestions on how to improve each of these components. Interviews were transcribed verbatim and (as necessary) translated into English. Data were managed using Dedoose, (Dedoose Version 5.0.11 et al. 2014) a qualitative software that allows for real-time access to a secured database by a number of people in an analysis team. In line with previous qualitative process evaluations of complex interventions (Hargreaves et al. 2010), we used an inductive-deductive approach. First interviews were coded for broad themes by four researchers, using a structured coding framework developed from topics covered in the interview guide. Next, a second stage of inductive coding allowed sub-themes to emerge. Process evaluation findings are used to suggest how these might help refine the intervention in a future trial.

### Statistical methods

For this baseline manuscript, we compared baseline characteristics between participants enrolled in the intervention and control sites using proportions for categorical variables and means or medians (as appropriate) for continuous variables. An assessment of the distribution (normally or skewed) of continuous variables was performed. Statistical tests for descriptive analyses included chi-square tests for categorical variables, and Wilcoxon rank-sum or t-tests and for continuous variables.

#### Analysis of outcome data

This pilot was not powered for formal significance testing of the intervention effect on primary health, behavioral and gender empowerment outcomes. Rather, analyses will focus on assessing the effects of the intervention on proximal mediating factors (Figure 2), and separately on primary outcomes. Analyses for mediating variables will generally treat scores as continuous measures. For example, food security will be measured using the Household Food Insecurity Access Scale (HFAIS) score at the 1-year visit; change in household economic indicators over the 1-year follow-up period will be measured by subtracting baseline from 1-year follow-up data for household expenditures, consumption and income (from agricultural and other sources). Initial comparisons will be based on group-specific descriptive summaries of observed outcomes and rank-based tests comparing outcomes between groups. We will also use regression methods to compare outcomes between groups and control for baseline characteristics. Analyses for primary health outcomes will proceed similarly, with appropriate choices of model for outcome type. For example, we will use pooled logistic regression for between-group comparisons of rates of viral load suppression, and mixed effects regression to compare changes in CD4 counts between groups. We will also make preliminary assessments of degree of mediation in models for primary outcomes via inclusion of mediating factors, with assessment of direct and indirect intervention effects of key mediating variables (Petersen et al. 2006). Finally, we will also perform intent-to-treat and per-protocol analyses of the pilot data, with the per-protocol subset limited to participants that obtained and used the intervention consistently over the 12-months of follow-up. Although these analyses will likely be underpowered for formal testing purposes, the resulting estimates and confidence intervals will be important for planning for the subsequent RCT.

## Results

### Screening and enrollment

Figure 3 describes study screening and enrollment numbers, along with reasons for participant ineligibility. We screened 142 and 154 adults, respectively at the intervention and control sites. We enrolled 72 and 68 participants at the intervention and control sites, respectively; four participants withdrew from the study, four from the intervention site and zero from the control site. One participant in the intervention arm failed to save the down payment necessary to receive the loan and was subsequently withdrawn from the study. The most common reasons for study ineligibility of screened participants included: did not have access to land or surface water, lived outside the study area, not on ART, and “other” reasons.

### Baseline characteristics of participants

At baseline (Table 2) participants at the intervention and control sites, respectively were similar in age (37 vs. 38 years), gender (51% female in both), education (19% vs. 18% completed secondary school) and marital status (75% vs. 79% married). Mental health and gender empowerment measures were similar among participants enrolled at the intervention and control sites. Reported monthly household income was lower among participants enrolled at the intervention in comparison to the control site (~$104) vs. (~$273), p = 0.008).

**Table 2:** **Comparison of baseline sociodemographic, economic, sexual risk behavior, dietary intake, clinical, mental health, gender empowerment, clinical and laboratory findings of participants enrolled in** ***Shamba Maisha*** **at the intervention and control sites**

	**Intervention**	**Control**	**P-value**
	**N = 72**	**N = 68**	
**Sociodemographic variables**	n (%)	n (%)	
Mean age in years (SE)	37 (0.80)	38 (0.80)	0.16
Female gender	37 (51)	35 (51)	0.92
Education ≥ secondary school	14 (19)	12 (18)	0.82
Current married	54 (75)	54 (79)	0.25
Mean number of people in household (SE)	6 (0.2)	7 (0.3)	0.12
**Food security level**			
Moderately food insecure	14 (20)	14 (21)	0.86
Severely food insecure	57 (80)	53 (78)	0.86
Body Mass Index (BMI) <18.5	13 (18)	5 (7)	0.054
**Economic indicators**			
Mean (SE) Monthly Household Income (USD*)	104 (21.3)	273 (53.1)	0.008
Land ownership (Self or Jointly)	60 (83)	49 (73)	0.14
**Sexual risk behavior**			
Any unprotected sex	13 (18)	12 (18)	0.95
Sex exchange (ever)	15 (21)	28 (41)	0.009
Sex exchange (last 3 months)	2 (3)	6 (9)	0.12
>1 Sexual partners in the past 3 months	9 (13)	9 (13)	0.92
**Dietary intake (frequency of consumption - times/week)**			
Grains mean (SE)	19.0 (0.8)	20.8(0.8)	0.1320
Vege`s mean (SE)	20.3 (0.9)	25.9 (0.7)	<0.0001
Fruit mean (SE)	9.0 (0.6)	10.6 (0.9)	0.1352
Meat, Poultry mean (SE)	3.4 (0.3)	5.3 (0.3)	<0.00001
Eggs mean (SE)	0.9 (0.1)	1.3 (0.2)	0.117
Dairy mean (SE)	1.9 (0.3)	4.5 (0.5)	<0.0001
Cooking fat mean (SE)	5.0 (0.3)	6.8 (0.1)	<0.00001
Sweets mean (SE)	7.1 (0.7)	11.9 (0.7)	<0.00001
**Mental health**			
Depression score ≥1.75 (clinical depression)	1 (1)	4 (6)	0.2
Mental health summary score (SE)	57.1 (3.4)	42.8 (5.6)	0.15
Mean internalized AIDS related stigma scale (SE)	14.1 (0.2)	13.7 (0.2)	0.19
Disclosed to husband/wife/partner	65 (97)	63 (95)	0.68
Mean Social Support Score (SE)	53.4 (10.8)	35.8 (8.3)	0.2
Problem drinking based on audit C	1 (1)	2 (3)	0.53
**Gender empowerment**			
Mean sexual relationship power scale (women only) (SE)	54 (10)	47 (8)	0.59
Mean decision making dominance scale (SE)	46 (5)	42 (4)	0.46
**Clinical, Laboratory and antiretroviral (ART) adherence**			
WHO clinical stage 3 or 4	15 (21)	22 (33)	0.12
Median CD4 (IQR) cells/mm^3^	446 (330–629)	475 (356–679)	0.33
Baseline CD4 ≤ 350 cells/mm^3^	20 (29)	16 (24)	0.467
Viral load above the limit of detection (≥20 copies/mL)	34 (49)	19 (28)	0.010
Physical health summary score (SE)	54.3 (3.6)	45.5 (4.0)	0.03
Hospitalized in the last three months	3 (4)	5 (7)	0.4
Reported ART adherence per Visual Analogue Scale < 90%	0 (0)	6 (9)	0.01

*Converted Kenyan shillings (Ksh) to US dollar (85 Ksh/$).

A similar proportion of participants at the intervention and control sites reported moderate and severe food insecurity based on the HFAIS, although a greater proportion of participants at the intervention site had a low BMI in comparison to participants at the control site (18% vs. 7%, p = 0.054) (Table 2). In regards to dietary intake, participants enrolled at the intervention site reported less frequency of vegetable, meat/poultry, dairy, cooking fat, and sweets than participants enrolled at the control site (Table 2).

Twenty-one percent and 33%, respectively of intervention and control site participants had WHO stage 3 or 4 disease. While median CD4 count was similar between arms (intervention: 446 cells/mm^3^ vs. control: 475 cells/mm^3^, p = 0.33), a greater proportion of participants enrolled at the intervention arm had a detectable HIV viral load than participants enrolled at the control arm (49% vs. 28%, respectively, p < 0.010); Self-reported ART adherence < 90% per visual analogue scale was rare in both arms of the trial (Table 2).

### Implementation successes and challenges

A variety of implementation successes and challenges were documented during the qualitative process evaluation. Positive aspects of *Shamba Maisha* included high acceptability during recruitment, successes with agricultural and financial training, and labor savings from using the pump. Implementation challenges included considerable concern about repaying loans, agricultural and irrigation challenges related to weather patterns, and a challenging partnership with the microfinance institution.

### Successes with *Shamba Maisha* implementation

1. *Strong willingness to join.* Nearly all participants interviewed expressed a strong interest in taking part in *Shamba Maisha*. Many had expectations that this intervention would improve their households and lives as expressed by one participant:

*What motivated me more to join the project was the training that they were coming to train us, they gave us some very good training. So when I heard about the type of training, the targets they put for us and the amount of money we would get…that if we got it then life would change for the better. (Male participant, 3 months, 31 years old).*

2) *Excellent agricultural training.* The agricultural portion of the training was a success and participants noted positive impacts even before they received the agricultural inputs, including increased and diversified crop production. The newly acquired farming skills were discussed with great pride by participants. The agricultural techniques resulted in larger harvests from smaller pieces of land – an outcome that many participants cherished:

*Before Shamba Maisha we just used to farm very huge pieces of land and get very small harvests. From the training we realized that we are able to farm a small piece of land and get a bumper harvest. This was really an eye opener for us. (Female participant, 3 months, 42 years old).*

Another woman explained crucial techniques around plowing, ridge planting, and nursery beds:

*We were taught on how to farm in order to get something good; for you to realize a good harvest, you need to plough deeply, then break it into more fine particles. Then you make ridges that we were given to help us in making the seedbeds and nursery. You make the nursery then plant them then later transfer then to the main farm. (Female participant, 3 months, 45 years old).*

Some participants even explained how neighbors were drawn to them as a result of the improved techniques:

*Because of Shamba Maisha, I was able to embrace a modern way of farming that has attracted people from all over to come and learn from it. (Male participant, 12 months, 44 years old).*

3) *Bolstering financial skills.* Some participants spoke of the utility of financial trainings, in particular the lessons around savings and record keeping:

*On savings we learnt a lot from the leader on the microfinance team on how we can save in the bank, we also learnt on record keeping while farming and how we can build our lives relying on the trainings…That was very good. (Male participant, 3 months, 38 years old).*

4) Ease of irrigation with water pumps. For a number of *Shamba Maisha* participants, the pump was easier to implement than previous irrigation strategies, such as bucket irrigation, leading to labor savings.

*I use the pump with a well. It helps in irrigation, at the times when these things need water I can use it. And I find it to be easier as compared to how I could have done it in the past where I could have taken maybe a bucket and use it to pour water. (Male participant, 3 months, 33 years old).*

*Before I used to fetch water from the borehole then I used a calabash to pour water directly. But now once I get two people, I just take the machine and pump water straight from the water source and irrigate just by pressing. So I use less energy in irrigation unlike before. (Male participant, 12 months, 44 years old).*

### Barriers to *Shamba Maisha* implementation

Despite these successes during implementation, participants noted a number of challenges, and had some recommendation for improving the intervention.

1. *Fear of loan repayment.* Some participants talked about the loan repayment as a potentially discouraging aspect of the intervention:

*We were going to be given loan and we’d be expected to repay some money back. This discouraged people and it’s still a factor that continue to discourage others…. here in Luo, they are scared of ‘debt recoveries”. (Male participant, 3 months, 43 years old).*

2) *Lack of choice in how to use loan. While many were satisfied with the commodities provided as part of Shamba Maisha,* some suggested that to improve the intervention, *Shamba Maisha* should provide the loan but allow people to decide how best to use it. For example, one man spoke about preference for livestock over farming:

*Farming, it is very difficult and requires you to take a lot of time, because people like us if you are doing it, it is something that you do full time. But we were thinking that if there was a different way other than farming, if one could choose what he could do then I think it could be better. … If I was told to choose then I would prefer to be given a cow, because that is something that I know and I have had some experience with. (Male participant, 3 months, 35 years old).*

Another participant wished for crops that were less perishable so that they can be stored and sold when the market was better:

*In my own view to help people more, if we could be introduced to a new type of farming or crop, that could uplift us because vegetables are little in terms of production and also are very perishable. So if you can introduce some new crops that even after harvesting can be stored until they fetch a good price in the market. You can keep them and later decide on the quantity to sell to repay the loan. (Female, 12 months, 40 years old).*

3) *Agricultural Challenges.* While agricultural productivity was reported to have improved by the majority of participants, variability in weather patterns impacted crop production throughout the course of the study period and across the communities. There were periods of flooding and hail storms which adversely affected crop production. For example, one woman complained of weather-related crop damage, despite describing having been very well trained to farm in the program “All my crops have been destroyed by the hailstorm and I have no means of repaying the loan.” (Female participant, 3 months, 38 years old) Rain gauges were used to monitor precipitation at the district hospitals in the intervention and control communities. Annual rainfall was reported to be higher than normal over the course of the one-year intervention with extended rainy seasons. As a result, the irrigation technology was not utilized as much as expected, decreasing some of the potential advantages.

*Irrigation is good, though we haven’t embraced it to an extent that it can fully benefit us because when we started it…the rain started. Our pumps are not being used because currently it’s rainy season and you can’t really sow vegetable seeds when there is too much rain. (Male participant, 12 months, 44 years old).*

4) *Pump Challenges.* Although most participants reported that the pumps were easy to use, some male and female participants had difficulty operating the pumps on their own.

As described by one participant:

*It is impossible to operate it comfortably as an individual. It demands that it is operated by two or three people with one person pumping the water as one directs the pipe to the shamba [farm]. When I used it, my sons had to assist me. (Female participant, 3 months, 30 years old).*

5) *Challenges with Microfinance Institution.* The local microfinance organization selected for the study had poor governance and was unable to fulfill the obligations of the study. Specifically, there were significant delays in dispersal of the loans and fiscal insolvency, leading to the closure of the local branch:

*It [The microfinance organization] could be closed for even 3 months. You go there and find the offices are closed and sometimes your money is in there…so it leaves you with a lot of doubts…And that is the main reason why many people ended up having fears that their money would get lost here. (Male participant, 3 months, 28 years old).*

Based on this finding, which emerged early in the process evaluation data collection, study leadership terminated the partnership with *Adok Timo* and transferred the loans to a local NGO which managed the loan collection for the remainder of the study period.

## Discussion

This pilot study was designed to determine the acceptability and feasibility of the intervention and control conditions, and to determine the preliminary impact of the intervention on mediating outcomes (food security, and household economic indicators). In the baseline evaluation of *Shamba Maisha*, we demonstrated that screening and enrollment into the intervention and control groups was rapid. Enrollment of the 140 participants took only four months, and the screening to enrollment ratio was similar between study arms. Thus, patients at the two study facilities were interested to screen and enroll in the study, i.e. enrollment into the control conditions did not appear to mitigate interest in study participation. This finding has important implications in regards to feasibility for advancing the intervention into a phase 3 cluster randomized controlled trial.

We found that the participants in the intervention and control groups were relatively similar demographically. However, participants at the control site reported a higher household income and dietary intake of vegetables and various protein sources. Importantly, participants in the control group were more likely to have an HIV-1 viral load below the limit of detection than participants enrolled in the intervention group. Self-reported ART non-adherence was uncommon in both groups. The lower proportion of non-detectable plasma viral loads in the intervention group may be related to the fact that the intervention group was economically worse off at baseline, and other studies have shown that markers of lower socioeconomic status are associated with delayed entry into care and worse HIV outcomes. In prior clinical programs in sub-Saharan Africa, viral load suppression ranges from 18% - 41% in observational studies (Hassan et al. 2014; Liegeois et al. 2012; Anude et al. 2013). Thus, our findings are relatively consistent with these results. Of note, this pilot study was not powered or designed to detect differences in viral load suppression by study arm. Rather the future trial will address variability and heterogeneity by randomizing approximately eight matched pair cluster (16 sites). We plan to match sites on a variety of criteria including sociodemographic factors, average rainfall, soil type, access to predominant source of surface water for irrigation (lake, vs. river, vs. shallow wells) and facility type (dispensary, vs. health center, vs. hospital).

Process evaluations garner in-depth knowledge around implementing complex interventions, which aids both interpretation of trial findings and informs potential replication (Oakley et al. 2006). Our process evaluation suggested robust acceptability of the *Shamba Maisha* intervention and trial, which aligns with the nearly 100% follow-up rate for both intervention and control participants at trial close. We also found that agricultural and financial training has strong advantages on their own, and that training should be integral to successful microfinance organizations. As anticipated, our qualitative data suggest that the microirrigation intervention can be laborsaving.

We also had significant challenges, most notably with regard to the microfinance component of the intervention. The partnering microfinance institution was unreliable, insolvent, and had a local branch closure during the trial. This is perhaps not surprising, given that past attempts to offer microfinance alongside HIV-related activities have experienced major challenges (Epstein 2006; Gregson et al. 2007). Researchers from the IMAGE intervention concluded that a strong microfinance partnership is indeed a pre-requisite to sustainably scaling this type of multisectoral intervention (Hargreaves et al. 2011). Thus, the replication of this trial in a larger setting should entail partnering with a microfinance institution that is financially stable and whose business model can accommodate *Shamba Maisha*. Participant concerns around loan repayment are an important ethical consideration; particularly in light of recent findings that microfinance may fail to provide enough income for financially disadvantaged households (Stewart et al. 2010; Barnes et al. 2001; Kaboski & Townsend 2008). As a result, we plan to consult local and global experts in microfinance in order to ensure that this component meets the needs of the study, and is designed with the potential for scale-up and sustainability is similar settings in East Africa.

These findings should be considered in light of design limitations. The pilot cluster-randomized design included two sites, and thus will need to be replicated in a larger cluster randomized trial. Second, qualitative process evaluations may suffer from respondent bias, particularly if participants held the impression that researchers were part of the intervention delivery team. We attempted to address this shortcoming by hiring separate in-depth interviewers that were not part of the primary study team. Lastly, the challenges with our microfinance organization made it difficult to assess the full potential of that component of the intervention.

We expect the pilot study will provide critical new evidence regarding the implementation of a multisectoral agricultural intervention aimed to improve food insecurity, household economic indicators and health among HIV-infected persons in rural Kenya. Following evaluation of the quantitative and qualitative results from this study, we plan to scale up this pilot to a cluster randomized trial to definitively assess the effectiveness of this intervention on the health of PLHIV using ART.

## Abbreviations

ART: Antiretroviral therapy
ARV: Antiretrovirals
AUDIT-C: Alcohol Use Disorders Identification Test
BMI: Body mass index
FACES: Family AIDS Care & Education Services
HFAIS: Household Food Insecurity Access Scale
KEMRI: Kenyan Medical Research Institute
IPM: Integrated pest and disease management
MOS-HIV: Medical Outcomes Study HIV Health Survey
MUAC: Mid upper arm circumference measurements
NGO: Non-governmental organization
ODK: Open Data Kit
PLHIV: People living with HIV/AIDS
RCT: Randomized controlled trial
SRPS: Sexual Relationship Power Scale
UCSF: University of California, San Francisco
